# Efficacy and safety of belumosudil for refractory chronic graft-versus-host disease in routine practice

**DOI:** 10.1007/s00277-026-06760-4

**Published:** 2026-02-04

**Authors:** Garret M.K. Leung, Joycelyn P.Y. Sim, Thomas S.Y. Chan, Carol Y.M. Cheung, Eric Tse, Albert K.W. Lie, Harinder Gill, Yok-Lam Kwong

**Affiliations:** Department of Medicine, Professorial Block, Queen Mary Hospital, Pokfulam Road, Hong Kong, China

**Keywords:** Belumosudil, Chronic graft versus host disease, Allogeneic hematopoietic stem cell transplantation, Ruxolitinib

## Abstract

Chronic graft versus host disease (cGVHD) affects 30–50% of allogeneic hematopoietic stem cell transplantation recipients, with those failing standard therapy including ruxolitinib constituting an important unmet medical need. We retrospectively analyzed the use of the ROCK2 inhibitor belumosudil in moderate to severe cGVHD that had failed ≥ 2 lines of systemic therapies, in order to evaluate its efficacy and safety in routine practice. Eighteen men and thirteen women at a median age of 50 (21–68) years with cGVHD (moderate, *N* = 12; severe, *N* = 19) that had failed a median of 3 (2–6) lines of systemic therapy were studied. Twenty-eight patients (90%) had failed prior ruxolitinib therapy, with seventeen patients (55%) continuing ruxolitinib despite unsatisfactory response. Among 29 patients evaluable, overall response rate was 52% (complete response:7%; partial response: 45%). Median time to response was 2.1 months (95% confidence interval, CI: 1.6–5.7), and was significantly shorter in patients older than 50 years (*P* = 0.008) and having received < 4 lines of therapy (*P* = 0.007). Median duration of response was 36.8 weeks (95% CI: 16.4 weeks to not reached). Median time to reduction of immunosuppressants by ≥ 50% was 6.7 months (95% CI: 2.3–12.2). Grade ≥ 3 adverse events occurred in 13% (4/31) of patients. Six patients (19%) discontinued treatment. At 12 months, overall survival and failure-free survival were 95% and 55%. Concomitant ruxolitinib significantly increased the probability of immunosuppressant reduction (*P* = 0.007). Belumosudil showed promising efficacy and safety in cGVHD patients in routine practice.

**Supplementary Information:**

The online version contains supplementary material available at 10.1007/s00277-026-06760-4.

## Introduction

Chronic GVHD (cGVHD) develops in 30–50% of allogeneic hematopoietic stem cell transplantation (HSCT) recipients [1–3], manifesting as heterogeneous, multi-organ involvement that causes substantial long-term morbidity [4]. Corticosteroids remain the first-line treatment [5–8], with nearly 50% of patients experiencing treatment failure within the first year [9]. Ruxolitinib, a JAK1/2 inhibitor, achieved a response rate of nearly 50% in steroid-refractory cGVHD [10], rendering it a standard second-line treatment. However, ruxolitinib failure occurred in 35–42% of cGVHD patients within 6–12 months [10, 11], representing a critical unmet need for effective treatment in these patients.

Belumosudil selectively inhibits Rho-associated coiled-coil-containing protein kinase 2 (ROCK2), a key regulator of immune cell differentiation and fibrotic pathways implicated in cGVHD pathogenesis [12, 13]. In pivotal trials of belumosudil in cGVHD, the overall response rate (ORR) was 62–77% after 1–5 prior lines of therapy, with a median duration of response (DOR) of 35–54 weeks [14–16]. These data were supported by retrospective studies of belumosudil in cGVHD, showing an ORR of 42–57% in heavily pretreated patients [17–19]. 

To date, real-world data on belumosudil in cGVHD remain limited, particularly in Asian populations. Furthermore, the efficacy of belumosudil in patients failing ruxolitinib has not been extensively defined. In this retrospective single-center study, we evaluated the safety and efficacy of belumosudil in cGVHD patients.

## Methods

###  Patients

Consecutive adult patients receiving more than one dose of belumosudil for the treatment of ongoing active cGVHD after allogeneic HSCT from January 2024 to March 2025 were included and retrospectively analyzed. There were otherwise no inclusion or exclusion criteria. Diagnosis, grading and response of cGVHD were based on the National Institute of Health (NIH) criteria [20, 21]. Patients had moderate or severe cGVHD, which had failed at least 2 prior lines of systemic treatment. Concurrent immunosuppressants (IST) were allowed.

### Treatment, response assessment and toxicity

Belumosudil was administered at a single daily dose of 200 mg (twice daily if a concurrent proton-pump inhibitor was used). Organ-specific severity (0–3), global severity (mild, moderate and severe), and response of cGVHD were determined by NIH criteria. 20,21 Responses were assessed after 4 weeks of treatment. In patients achieving complete response (CR), partial response (PR), or stable disease (SD), belumosudil was continued until loss of response or unacceptable toxicity. Continuation of belumosudil beyond disease progression was permitted at the discretion of the attending physicians when there was perceived clinical benefit without grade ≥ 3 toxicities. Survival outcomes were analyzed by an intention-to-treat basis for the entire cohort. This study was approved by the Institutional Review Board of the Hong Kong West Cluster/University of Hong Kong.

### **Endpoints**

The primary endpoints were ORR (CR + PR) and adverse events, which were graded according to the Common Terminology Criteria for Adverse Events (CTCAE) Version 5.0 [22]. Secondary endpoints included time to response (TTR), defined as time from belumosudil initiation to first documented CR or PR lasting ≥ 4 weeks; DOR, defined as time of first response to cGVHD progression, dose escalation of concurrent systemic therapy, or initiation of new systemic cGVHD treatment; and time to 50% IST reduction (TTIR), defined as time from belumosudil commencement to either ≥ 50% dose reduction of each concurrent IST, or ≥ 50% reduction in total number of concurrent IST excluding belumosudil.

### Survivals

Overall survival (OS) was defined as time from treatment to death from any cause. Failure-free survival (FFS) was defined as time from treatment to disease relapse, cGVHD progression, escalation of concurrent systemic therapy, new systemic cGVHD treatment, unacceptable treatment toxicity, or death. All survivals were censored at the time of last follow-up.

### Statistical methods

Quantitative variables were compared using Pearson’s Chi-square or Fisher’s exact tests. TTR, DOR, TTIR, OS and FFS were analyzed by the Kaplan-Meier method, with potential impacting variables compared with the log-rank test. Hazard ratios (HR) were calculated with the Cox proportional hazards regression model. Variables showing a significant association (*P* < 0.05) in univariate analyses were included in multivariate analyses. Statistical analyses were performed with the R-4.4.1 statistical software (http://cran.r-project.org/).

## Results

### Patients

Thirty-one patients, comprising 30 Asians and 1 Caucasian, were treated with belumosudil (Table 1). The median follow-up time was 9.4 months (range, 0.4–14.8 months). The median age was 50 years for recipients (range: 21–68 years) and 38 years for donors (range: 18–63 years). Recipients were predominantly men (58%), while donors were more frequently women (58%). Sex-mismatched transplantations were common (female to male: 39%; male to female: 23%). Underlying diseases included acute myeloid leukemia (42%), non-Hodgkin lymphoma (19%), myelodysplastic neoplasms/myeloproliferative neoplasms (19%), acute lymphoblastic leukemia (13%), and chronic myeloid leukemia (7%). Donors were primarily matched unrelated (42%) or matched sibling (26%), with mismatched unrelated and haploidentical donors each constituting 16%. Peripheral blood was the exclusive HSC source (100%). Cytomegalovirus (CMV) seropositivity was high in both recipients (87%) and donors (74%), with most pairs being positive-positive (71%). Conditioning intensity was myeloablative in 65% and reduced-intensity in 35% of cases. GVHD prophylaxis was calcineurin inhibitor-based (cyclosporine, short-course methotrexate, mycophenolate mofetil) in 81% of patients, and post-transplantation cyclophosphamide, cyclosporine and mycophenolate mofetil, in 19% of patients. A small proportion (6.5%) had a prior allogeneic HSCT.

**Table 1:** Clinicopathologic features of 31 patients with chronic graft versus host disease treated with belumosudil

Clinicopathologic features	Number (%)
Race	
Asian	30 (97%)
Caucasian	1 (3%)
Age (years)	
Recipient – median (range)	50 (21–68)
Donor – median (range)	38 (18–63)
Male sex	
Recipient	18 (58%)
Donor	13 (42%)
Disease	
Acute myeloid leukemia	13 (42%)
Non Hodgkin lymphoma	6 (19%)
Myelodysplastic/myeloproliferative neoplasms	6 (19%)
Acute lymphoblastic leukemia	4 (13%)
Chronic myeloid leukemia	2 (7%)
Prior transplantation	
Autologous	0 (0%)
Allogeneic	2 (6.5%)
Donor type	
Matched sibling	8 (26%)
Matched unrelated	13 (42%)
Mismatched unrelated	5 (16%)
Haploidentical	5 (16%)
Donor-recipient sex match	
Female to female	6 (19%)
Female to male	12 (39%)
Male to female	7 (23%)
Male to male	6 (19%)
Hematopoietic stem cell source	
Peripheral blood	31 (100%)
Bone marrow	0 (0%)
Cytomegalovirus seropositivity	
Recipient	27 (87%)
Donor	23 (74%)
Donor-recipient cytomegalovirus serology match	
Negative to negative	3 (10%)
Negative to positive	1 (3%)
Positive to negative	5 (16%)
Positive to positive	22 (71%)
Conditioning intensity	
Myeloablative	20 (65%)
Reduced intensity	11 (35%)
Graft versus host disease prophylaxis	
Conventional calcineurin inhibitor-based	25 (81%)
Post-transplantation cyclophosphamide	6 (19%)

### **Characteristics of cGVHD**

Antecedent acute GVHD of grade 2–4 occurred in 12 patients (39%) (Table 2). The median time of onset of cGVHD was 8 months (range, 4–29 months) after allogeneic HSCT. Most patients had severe cGVHD (61%, N = 19), with the rest having moderate disease (39%, N = 12). The most frequently involved organs were eyes (52%, N = 16), mouth (39%, N = 12), and lungs (39%, N = 12). Five patients had a forced expiratory volume in 1 s (FEV1) of ≤ 39% or an NIH lung symptom score of 3 before initiation of belumosudil treatment. Patients were heavily pretreated (prior lines of systemic therapies: two, N = 9, 29%; three, N = 8, 26%; four, N = 9, 29%; five, N = 3, 10%; six, N = 2, 6%). All patients had received prior corticosteroids, which were tapered off due to side effects. Concurrent IST were common (N-29, 94%) (Table 2). Importantly, 90% (N = 28) of patients were considered to have failed prior ruxolitinib; with 45% (N = 11) already having discontinued ruxolitinib owing to lack of efficacy, and 55% (N = 17) continuing ruxolitinib at a median dose of 10 mg daily (range, 5–20 mg) despite unsatisfactory response. Hence, all patients were considered to have active cGVHD despite on-going therapy. Belumosudil was initiated at a median time of 56 months (range, 8–176 months) post-HSCT.

**Table 2:** Characteristics of chronic graft versus host disease (cGVHD) in 31 patients treated with belumosudil

Characteristics	Number
Time from HSCT to belumosudil treatment, months (range)	56 (8,176)
National Institute of Health cGVHD severity	
Moderate	12 (39%)
Severe	19 (61%)
Prior acute GVHD	
Grade 2–4	12 (39%)
Grade 3–4	2 (7%)
Organ involvement	
Eyes	16 (52%)
Mouth	12 (39%)
Lungs	12 (39%)
Joint and fascia	8 (26%)
Skin	5 (16%)
Liver	5 (16%)
Gut	1 (3%)
Prior lines of therapy	
2	9 (29%)
3	8 (26%)
4	9 (29%)
≥5	5 (16%)
Prior corticosteroids	31 (100%)
Prior ruxolitinib	28 (90%)
Concurrent immunosuppressants	
≤2	21 (68%)
3	5 (16%)
≥4	3 (10%)
Concomitant systemic cGVHD therapy type	
Ruxolitinib	17 (55%)
Corticosteroids	5 (16%)
Cyclosporine	9 (29%)
Sirolimus	3 (10%)
Mycophenolate mofetil	10 (32%)
Ibrutinib	3 (10%)
Thalidomide	3 (10%)
Imatinib	1 (3%)

### Treatment response

**Fig. 1:**
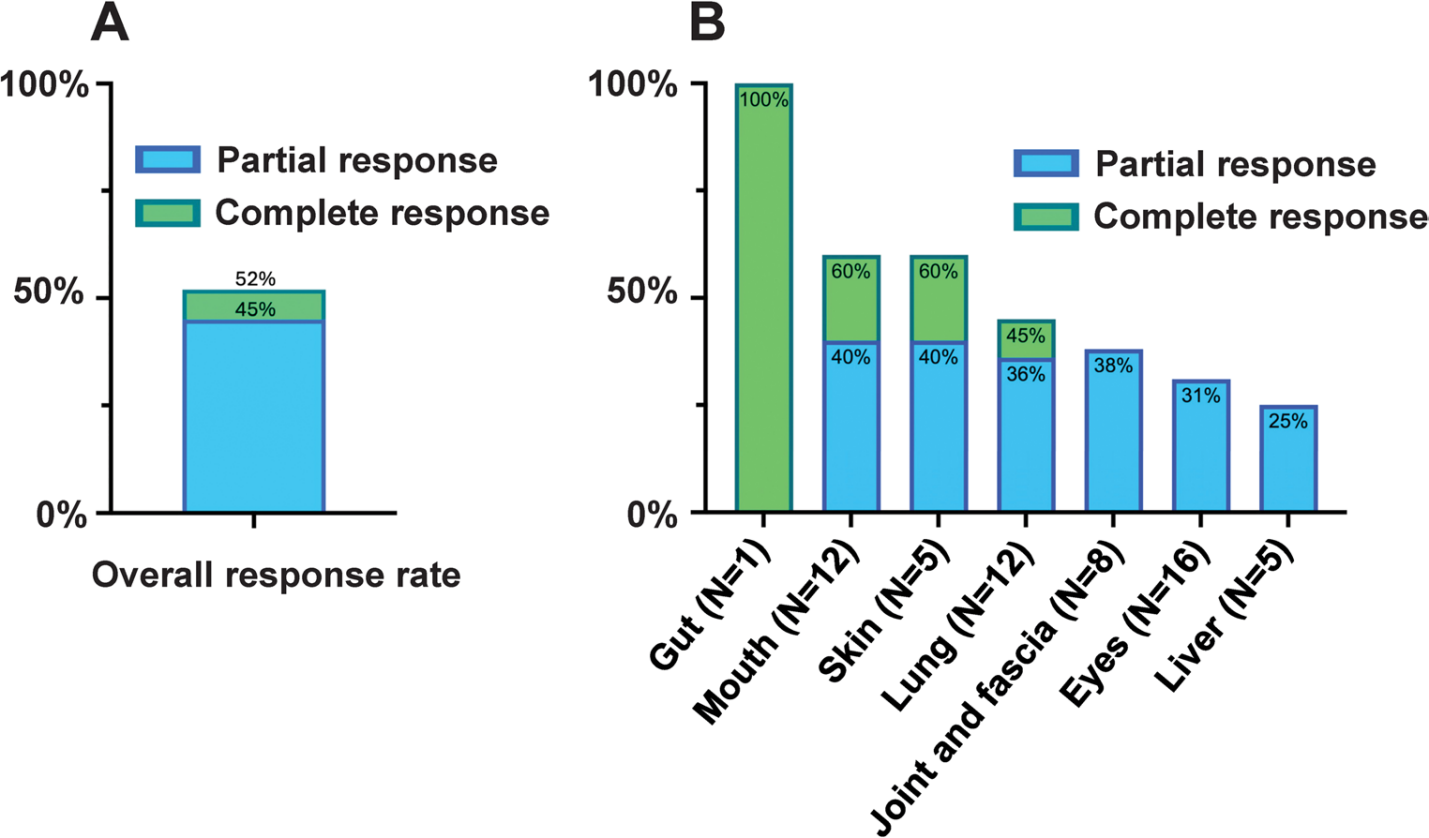
Response of chronic graft versus host disease to belumosudil. **A**. Overall response. CR: complete response; PR: partial response, **B**. Organ-specific response

Among 29 cases treated with belumosudil for more than one month and hence considered evaluable, 15 patients responded, giving an ORR of 52%. The best response achieved was CR in 2 patients (7%), and PR in 13 patients (45%) (Fig. 1 A). In patients receiving concomitant ruxolitinib, the ORR was 53% (CR: 12%; PR: 41%). Organ-specific cGVHD responses varied. Mouth and skin showed the highest ORR at 60% (CR: 20%; PR: 40%). Among patients with lung involvement, the ORR was 45% (CR: 9%; PR: 36%); and for the subgroup with NIH lung symptom score 3, ORR was 40%, all being PR. Lower response rates were observed for joint and fascia (PR: 38%), eye (PR: 31%), and liver (PR: 25%); with no CR achieved in these organs. The single patient with gut cGVHD achieved a CR (Fig. 1B).

### Kinetics of response

**Fig. 2:**
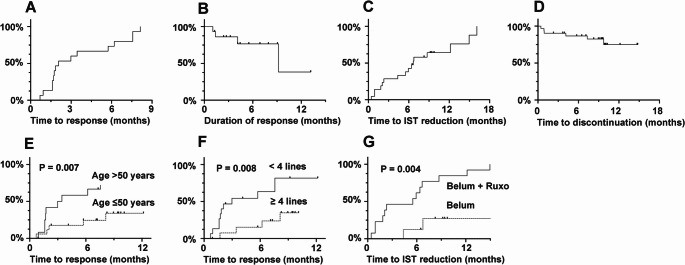
Kinetics of Response of chronic graft versus host disease. **A**. Time from belumosudil initiation to first response (median: 2.1 months, 95% confidence intervals: 1.6, 5.7). **B**. Duration of response after achieving partial response/complete response (median: 9.2 months, 95% confidence intervals: 4.1, not reached). **C**. Time to 50% dose reduction of concurrent immunosuppressants (median: 6.7 months, 95% confidence intervals: 2.3, 12.2). **D**. Discontinuation of belumosudil (median discontinuation time: not reached) (6-month continuation rate: 87%, 95% confidence intervals: 75, 99) (12-month continuation rate: 75%, 95% confidence interval: 58, 97). **E**. Time from belumosudil initiation to first response, showing that patients > 50 years old had significantly faster response than those ≤ 50 years old. **F**. Time from belumosudil initiation to first response, showing that patients receiving < 4 lines of therapy had significantly faster response that those receiving ≥ 4 lines of therapy. **G**. Time to 50% dose reduction of concurrent immunosuppressants, showing that those receiving combined belumosudil (belum) and ruxolitinib (ruxo) treatment had significantly shorter time to 50% dose reduction than those receiving belumosudil alone

Responses to belumosudil improved over time. At 1 month, only 7% (2/29) of patients achieved CR or PR. By 3 months, ORR increased to 32% (9/28), increasing further to 38% (9/24) at 6 months and 50% (10/20) at 9 months (Table 3). Among the 15 responding patients, the median TTR was 2.1 months (95% confidence interval, CI: 1.6–5.7 months) (Fig. 2 A). Four patients lost response after 1.1 to 9.2 months. The median DOR was 36.8 weeks (95% CI: 16.4 weeks to not reached) (Fig. 2B). The estimated median TTIR in patients achieving CR, PR, or SD was 6.7 months (95% CI: 2.3–12.2 months) (Fig. 2 C).

**Table 3:** Response of chronic graft versus host disease to belumosudil at different timepoints

	1 month	3 months	6 months	9 months	12 months
Patients	*N* = 29	*N* = 28	*N* = 24	*N* = 20	*N* = 3
Complete or partial response	2 (7%)	9 (32%)	9 (38%)	10 (50%)	1 (33%)
Stable disease	26 (90%)	17 (61%)	11 (46%)	7 (35%)	1 (33%)
Disease progression	1 (3%)	2 (7%)	4 (17%)	3 (15%)	1 (33%)

### Safety and discontinuation

Grade ≥ 3 AEs were uncommon and occurred in only four patients (4/31, 13%) (infections, N = 2; transaminitis, N = 1; thrombocytopenia, N = 1). Three of them received combined ruxolitinib belumosudil treatment (odds ratio: 2.79; 95% CI: 0.256–30.3; P = 0.61). Six patients discontinued belumosudil because of intolerable side effects (rash, N = 1; malaise, N = 1, dysgeusia, N = 1), disease progression (liver cGVHD, N = 1), on-going infection (pulmonary aspergillosis, N = 1) and death unrelated to treatment (intracranial hemorrhage, N = 1). Importantly, 87% and 75% of patients were continued on belumosudil at 6 and 12 months without any other grade ≥ 3 AEs (Fig. 2D).

### Survivals

**Fig. 3:**
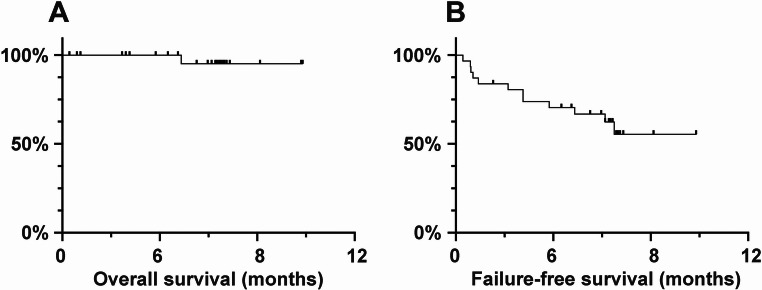
Survivals of patients with chronic graft-versus-host disease treated with belumosudil. **A**. Overall survival, **B**. Failure-free survival

There were no relapses of the underlying primary diseases during the study period. One patient died of an unrelated intracranial hemorrhage. The OS was 100% and 95% at 6 and 12 months (Fig. 3 A). The median FFS was not reached. The 6-month and 12-month FFS was 70% and 55% (Fig. 3B).

### Prognostic indicators

The ORR was significantly higher in patients aged > 50 years as compared with those age ≤ 50 years (77% versus 31%; OR: 6.77; 95% CI: 1.10–56.5; *P* = 0.025); and in patients receiving < 4 prior lines of systemic cGVHD therapy as compared with those receiving ≥ 5 lines (73% versus 29%; OR: 6.36; 95% CI: 1.07–47.9; *P* = 0.027) (Supplementary Table 1). Univariate analysis showed that shorter TTR was associated with age > 50 years (hazard ratio, HR: 4.21, 95% CI: 1.40–12.7; *P* = 0.011), with a 12-month cumulative rate of 89% (*versus* 35% in those aged ≤ 50 years) (*P* = 0.007) (Fig. 2E); and < 4 prior lines of therapy (HR: 4.33, 95% CI: 1.35–13.8; P = 0.014), with a 12-month rate of 82% (*versus* 35% in ≥ 5 lines of therapy) (*P* = 0.008) (Fig. 2 F). However, these associations lost significance in multivariate analysis. Notably, concurrent ruxolitinib use was associated with a higher rate of achieving ≥ 50% dose reduction of concurrent IST (HR: 5.62; 95% CI: 1.60–19.7; *P* = 0.007), with 12-month rates of 85% *versus* 27% in patients not receiving concomitant ruxolitinib (*P* = 0.004) (Fig. 2G). No factors significantly influenced DOR or FFS (Supplementary Table 2).

## Discussion

Our findings represented the real-world efficacy and safety of belumosudil in the treatment of cGVHD in patients failing at least 2 prior systemic lines of therapy. Importantly, most patients had failed ruxolitinib. We observed a time-dependent gradual improvement of response, with the best responses (CR + PR) achieved nine months after initiation of belumosudil. Compared with the pooled analysis of 208 patients in the belumosudil pivotal studies [15], we observed a lower ORR of 52% (*versus* 72% in pivotal studies) and a shorter DOR of 36.8 weeks (*versus *62.3 weeks in pivotal studies); which might be related to differences between the patient cohorts studied. Firstly, 90% of our cohort had prior ruxolitinib failure as compared with only 27% in the pivotal studies [15], suggesting that our cases had more refractory diseases. Secondly, although 94% of our patients received concomitant IST, only 16% received corticosteroids. This contrasts with the belumosudil pivotal studies, in which 97% of patients had concomitant corticosteroids, which could possibly have contributed to their higher response rates. Thirdly, only one patient in our study had gut involvement. In contrast, registration studies (KD025-208 and ROCKstar) recruited a much higher proportion of patients with gastrointestinal involvement (upper: 15%; lower: 9%) [15]. As belumosudil induced the most favorable response in gut cGVHD (ORR for upper and lower gastrointestinal tract: 61% and 72%) [15], a higher proportion of patients with gut involvement in registration studies would also contribute to an apparently higher ORR [15]. Fourthly, Asian patients, who constituted 97% of our cohort, were severely under-represented in the pivotal studies (2%, 3/132) [14], so that racial differences in response remain undefined. To address this data gap, two prospective Asian studies comprising 51 patients have recently been reported [23, 24]. However, these patients had failed merely ≥ 1 line of systemic therapy [23], with 20 subjects in one study not even having received ruxolitinib before [24]. In these patients with less refractory cGVHD, the ORR varied from 73.3% to 85.7% [23, 24]. These response rates were similar to that of 73% observed in our study for patients receiving < 4 lines of therapy. Hence, a key observation in our study and others [23, 24] is early use of belumosudil in the treatment of cGVHD may maximize response. The large number of prior lines of therapy (70% of patients had received ≥ 3 lines) was due to the fact that patients were treated outside of a clinical trial, so that access to belumosudil was not timely and only possible via a compassionate program or after its approval and registration. Another observation in this study was that patients > 50 years old showed a better ORR. This finding contrasts with those of other reports, which suggested that patient age did not have a significant impact on treatment response [14, 15, 17, 18]. Because our observations were made in a relatively small cohort of patients, they ought to be validated in future cohort of patients, particularly in routine clinical practice.

Despite a lower ORR, 1-year FFS was similar in our patients as comparable with those of the pivotal studies (55% *versus *56%) [15], which might reflect that in our cohort, fewer patients lost responses and there was no relapse of the primary diseases and merely one unrelated death. Given that a much higher proportion (55%) of our patients received concomitant ruxolitinib as compared with those in the pivotal studies (only 1%) [15], it remains important to define if ruxolitinib might act synergistically with belumosudil.

Additionally, our study addressed a critical unmet need in pulmonary cGVHD, a significant contributor to non-relapse mortality after allogeneic HSCT [25]. Despite the use of newer agents, response rates of pulmonary cGVHD remain poor at 9% with ruxolitinib [10] and 14% with ibrutinib [26]. While the belumosudil registration trial showed an ORR of 26% (CR: 13%) in pulmonary cGVHD, it only enrolled low-risk patients and excluded those with the highest risks, viz. FEV1 ≤ 39% or NIH lung symptom score 3, precisely the population most in need of effective treatment [14]. In our real-world cohort, we observed an encouraging ORR of 45% (CR: 9%; PR:36%) in pulmonary cGVHD. Particularly, we treated five patients with severe pulmonary cGVHD (NIH lung symptom score of 3), cases typically excluded from the belumosudil pivotal studies, achieving a PR of 40%. The responses in these patients were assessed by NIH symptom scoring. Although serial spirometry was also performed, the small number of patients with lung cGVHD in our cohort precluded meaningful statistical analysis. It will be interesting for future studies to assess whether spirometry offers a more accurate or timely reflection of response. Another approved agent, axatilimab, showed a promising ORR of 47% in pulmonary cGVHD [27]. However, it is currently unavailable in Asia. Hence, belumosudil use in pulmonary cGVHD should be further evaluated and compared with other available agents.

Given the favorable safety profile of belumosudil, combination strategies have been explored in retrospective studies [28–31]. In our study, the additional use of belumosudil in seventeen patients who did not respond to ruxolitinib but were nevertheless still receiving it led to an ORR of 53%. This salvage rate was comparable with the reported ORR of combined belumosudil and ruxolitinib at 42–55% [30, 31]. A recent retrospective study of 26 patients suggested that the ORR across most organ systems, except skin and gut, was higher when belumosudil was combined with ruxolitinib as compared with other agents (prednisolone, tacrolimus, or sirolimus) [29]. Specifically, in pulmonary cGVHD, the belumosudil-ruxolitinib combination showed a superior ORR compared with other combinations (50% *versus *17%) [29]. However, another recent study, while demonstrating a high ORR of 52%, did not find improved outcome with combined ruxolitinib belumosudil as compared with belumosudil monotherapy, although patients on combination therapy had more severe cGVHD [32]. Interestingly, in our study, the belumosudil-ruxolitinib combination, while not impacting on ORR, DOR, TTR, or FFS, significantly increased the probability of achieving 50% dose reduction of other IST (HR 5.62, *P* = 0.007). This observation aligned with recent evidence showing that other IST could be tapered or discontinued in all responding patients receiving the belumosudil-ruxolitinib combination therapy [30]. Importantly, our study and others [29, 30] showed that the belumosudil-ruxolitinib combination was well tolerated, with adverse events consistent with those expected for cGVHD patients receiving IST.

Finally, previous prospective and retrospective studies showed that approximately a third to over a half of the patients receiving belumosudil could achieve ≥ 50% reduction of corticosteroid dose, but whether non-steroidal IST could be dose-reduced remained undefined [16, 17, 24]. A unique finding in our study was that steroid and non-steroidal IST could all be tapered during belumosudil treatment, with a median TTIR of 6.7 months. This finding has clinical significance, because prolonged use of IST increases the risks of infection and attenuates graft-versus-malignancy effects [33, 34]. 

This study provided a real-world analysis of belumosudil in the management of highly refractory cGVHD. Key strengths included treatment of patients with features/severity of cGVHD that were excluded from registration trials, comprehensive organ-specific response assessment, and quantification of time to IST reduction, a clinically relevant endpoint. Limitations included the single-center retrospective nature, and short median follow-up. Future multicenter studies with larger cohorts are needed to validate our findings.

We conclude that belumosudil was safe and efficacious in Asian cGVHD patients with predominantly ruxolitinib-refractory disease. Its activity in severe cGVHD addressed critical unmet needs. Further studies to optimize sequencing and combination approaches involving belumosudil in advanced cGVHD are warranted.

## Supplementary Information

Below is the link to the electronic supplementary material.

Supplementary Material 1

## Data Availability

De-identified data are available from the corresponding author on reasonable request.
