# Supplementary material for: Efficacy and safety of belumosudil for refractory chronic graft-versus-host disease in routine practice
Source: Ann Hematol. 2026 Feb 4;105(3):84. doi: 10.1007/s00277-026-06760-4 (PMC12872659; doi:10.1007/s00277-026-06760-4)
Supplement: Supplementary file 1 — Supplementary Material 1 [file 277_2026_6760_MOESM1_ESM.docx]

**Supplementary Table 1. Prognostic Indicators for overall response rate**

| Clinicopathologic features | Odds Ratio | P value |
| --- | --- | --- |
|  |  |  |
| Age  Recipient >50 years  Donor >30 years | 6.77 (1.10, 56.5)  1.74 (0.17, 24.2) | 0.025  0.651 |
| Male sex  Recipient  Donor | 1.48 (0.27,8.32)  0.68 (0.12, 3.65) | 0.715  0.715 |
| HLA-mismatched | 0.66 (0.10,4.17) | 0.700 |
| Reduced intensity conditioning | 2.13 (0.37, 13.8) | 0.450 |
| Post-transplantation cyclophosphamide | 2.12 (0.25, 27.9) | 0.651 |
| Severe chronic graft versus host disease | 2.65 (0.46, 17.5) | 0.264 |
| <4 prior lines of therapy | 6.36 (1.07, 47.9) | 0.027 |
| <3 organs involved | 0.12 (0.00, 1.29) | 0.080 |
| Concurrent immunosuppressants | 0.00 (0.00, 2.49) | 0.224 |
| Concurrent ruxolitinib | 1.12 (0.20, 6.28) | >0.999 |
|  |  |  |

# **Supplementary Table 2.** **Cox-regression analysis of the impacts on clinical factors on response kinetics.**

| Duration of response | | |  |  |  | Time to response | | |  | |  |  |
| --- | --- | --- | --- | --- | --- | --- | --- | --- | --- | --- | --- | --- |
|  | **HR (Univariate)** | **P value** | | |  | **HR (Univariate)** | **P value** | **HR (Multivariate)** | | **P value** | | |
| Age  Recipient >50 years  Donor >30 years | 0.58 (0.08, 4.19)  0.73 (0.06, 9.10) | 0.586  0.808 | | |  | 4.21 (1.40, 12.7)  1.41 (0.32, 6.28) | 0.011  0.649 | 2.81 (0.88, 8.96) | | 0.080 | | |
| Male sex  Recipient  Donor | 0.76 (0.11, 5.44)  1.09 (0.15, 7.82) | 0.784  0.933 | | |  | 1.10 (0.39, 3.10) | 0.855 |  | |  | | |
| HLA-mismatched | 6.37 (0.58, 70.4) | 0.131 | | |  | 0.66 (0.21, 2.08) | 0.477 |  | |  | | |
| Reduced intensity conditioning | 2.37 (0.21, 26.2) | 0.481 | | |  | 1.97 (0.71, 5.45) | 0.194 |  | |  | | |
| Post-transplantation cyclophophamide | 6.37 (0.58, 70.4) | 0.131 | | |  | 1.45 (0.46, 4.57) | 0.524 |  | |  | | |
| Severe cGVHD | 0.72 (0.06, 8.03) | 0.791 | | |  | 1.43 (0.45, 4.54) | 0.539 |  | |  | | |
| Prior LOT <4 | 3.73 (0.26, 53.0) | 0.330 | | |  | 4.33 (1.35, 13.8) | 0.014 | 2.93 (0.86, 10.0) | | 0.087 | | |
| <3 organs involved | 5.91 (0.81, 43.1) | 0.136 | | |  | 0.45 (0.16, 1.27) | 0.132 |  | |  | | |
| Concurrent immunosuppressants | 1.23 (0.12, 12.9) | 0.861 | | |  | 0.30 (0.08, 1.09) | 0.068 |  | |  | | |
| Concurrent ruxolitinib | 2.06 (0.21, 20.1) | 0.534 | | |  | 1.37 (0.48, 3.91) | 0.561 |  | |  | | |
|  |  |  | | |  |  |  |  | |  | | |

| Time to 50% reduction of immunosuppressants | | |  |  |  | Failure-free survival | |  |
| --- | --- | --- | --- | --- | --- | --- | --- | --- |
|  | **HR (Univariate)** | **P value** | | |  | **HR (Univariate)** | **P value** | |
| Age  Recipient >50 years  Donor >30 years | 1.19 (0.45, 3.11)  0.54 (0.17, 1.68) | 0.726  0.284 | | |  | 0.83 (0.26, 2.62)  2.01 (0.26, 15.7) | 0.745  0.507 | |
| Male sex  Recipient  Donor | 1.12 (0.44, 2.84)  1.17 (0.46, 2.99) | 0.819  0.742 | | |  | 1.40 (0.42, 4.65)  0.38 (0.10, 1.41) | 0.585  0.147 | |
| HLA-mismatched | 0.82 (0.28, 2.36) | 0.708 | | |  | 1.78 (0.55, 5.69) | 0.333 | |
| Reduced intensity conditioning | 1.88 (0.74, 4.82) | 0.187 | | |  | 0.60 (0.16, 2.23) | 0.448 | |
| Post-transplantation cyclophosphamide | 0.80 (0.23, 2.84) | 0.735 | | |  | 0.95 (0.21, 4.43) | 0.953 | |
| Severe chronic graft versus hosts disease | 1.53 (0.50, 4.70) | 0.461 | | |  | 0.65 (0.20, 2.07) | 0.466 | |
| <4 prior lines of therapy | 1.99 (0.76, 5.18) | 0.159 | | |  | 1.21 (0.38, 3.87) | 0.748 | |
| <3 organs involved | 0.89 (0.33, 2.41) | 0.818 | | |  | 5.03 (0.65, 39.2) | 0.123 | |
| Concurrent immunosuppressants | 1.30 (0.17, 10.0) | 0.801 | | |  | 1.43 (0.18, 11.1) | 0.732 | |
| Concurrent ruxolitinib | 5.62 (1.60, 19.7) | 0.007 | | |  | 0.73 (0.23, 2.29) | 0.586 | |
|  |  |  | | |  |  |  | |

HR: hazard ratio
